# Hypertrophy of the Lingual Tonsil

**Published:** 1896-06

**Authors:** Mary E. Dickinson

**Affiliations:** Dansville, N. Y.


### HYPERTROPHY OF THE LINGUAL TONSIL.

BY MARY E. DICKINSON, M. D., Dansville, N. Y.

AT TIMES there have come under the observation of every medical practitioner patients with a history of marked tendency to "sore throat" upon slight exposure, a sense of fatigue in the throat after talking or singing a short time, hoarseness, a feeling that crumbs or other small particles are caught somewhere in the throat, sudden choking while eating due to food getting in the larynx, and the like. A dry cough sometimes accompanies these symptoms. This condition may have existed for years as a constant annoyance, increasing in severity until the aggravation compels the sufferer to seek relief.

Upon a general inspection there is seen nothing to warrant the trouble complained of. The hard and soft palate, uvula, pillars of fauces and pharynx seem to be in good condition. The tonsils may be somewhat affected, but not necessarily so.

What is the cause of all this trouble ?

Take a laryngoscope and see what you find. In its normal condition the base of the tongue and the part just above present an irregular uneven surface, due to the abundant distribution of papillæ and the layers of lymphatic tissue, embedded in which are the numerous mucous glands. This surface also has an abundant blood supply.

From the nature of the structure it is easy to see how it readily becomes the seat of hypertrophic changes. An ordinary amount of hypertrophy in this region is common and gives rise to no special discomfort. It is when the formation of this lymphatic tissue has filled up in some measure the glosso-epiglottic fossa, sometimes to encroachment upon the epiglottis itself, that the symptoms described are noticed. The blood-vessels often become varicosed and can be easily seen as red threads or lines through this hypertrophied tissue.

As is natural to expect, this condition is quite prevalent among speakers, teachers, clergymen and singers. It is found more among women than among men.

General treatment is of little use. The only help is in the reduction of this redundant growth. Some good may be obtained by painting the part with strong astringents, nitrate of silver or tincture of iodine, but this relief lasts only as long as the applications are continued.

The radical methods are removal of the tissue by use of the bistoury, curved scissors, cold snare or galvano-cautery.

Owing to the abundant blood supply often found in this condition the greatest care must be exercised in handling the cutting instruments.

The snare is always safe and desirable when the follicles are prominent and easily engaged in the loop, the hemorrhage following being of comparatively little account.

In my experience the use of the galvano-cautery has been most satisfactory. A very small portion of the affected area is treated at one sitting. This is painted with an 8 per cent. solution of cocaine until there is no sense of feeling in the part. In some instances, when the patient is unpleasantly affected by any application of cocaine, I use a fifty per cent. solution of carbolic acid, touching only the exact portion I wish to cover with the cautery.

The wire is bent to fit the curve of the tongue so that the cautery point may lie flat and close upon the surface treated. The cautery tip, moreover, is introduced cold, the interrupter in the handle giving this advantage, as well as that of withdrawing the tip with the current cut off.

These precautions obviate the risk of injury to the adjacent parts, especially the epiglottis, which danger is counted one of the strong objections to the use of the galvano-cautery in these cases. The number of applications of the point at one sitting depends upon the condition of the part, and is usually two, never more than three, covering a narrow area about the width of the point.

The objections stated to the use of this instrument are that the constitutional disturbances are apt to be severe, as nausea, diarrhea, fever, headache, loss of appetite, intense pain in the throat, etc. Perhaps such consequences would follow were a large portion or the whole of the affected tissue treated at one time, but in an experience including over one hundred cases I have not found these objectionable results. The nearest approach was when at the expressed desire of the patient I cauterised about three times the proper amount at one sitting, and only limited time would induce me to repeat such a treatment.

It is important to take plenty of time, at least six weeks, preferably three months, allowing from six to ten days' interval between the sittings.

There will be some discomfort for two days, especially when the cauterising is made on the sides of the tongue. The patient then may complain of pain in the ear on that side, the irritation extending along the stylo-glossus muscle. Hot wet applications to that part of the neck and face will afford the needed relief and may be continued as long as the pain lasts. If necessary give at bedtime the first night : phenacetine, gr. vj.; sulfonal, gr. xv. Often the patient need not change his usual diet or routine of life in any way.

The results from the obliteration of this hypertrophied tissue are very gratifying. Talking or singing are resumed normally with no sense of "tired throat," hoarseness or "sore throat," as before ; and there is no more coughing or choking at table. One patient who indulged in a sore throat with every adverse wind was called away after but part of the work was done. She returned with the experience of having had but one-half of a sore throat, the demarcation being clear enough, as she expressed it, to define with a pencil.

